# Supplementary material for: Comprehensive Investigation of Die-Back Disease Caused by Fusarium in Durian
Source: Plants (Basel). 2023 Aug 24;12(17):3045. doi: 10.3390/plants12173045 (PMC10490359; doi:10.3390/plants12173045)
Supplement: Supplementary file 1 [file plants-12-03045-s001.zip › Table-S1.pdf]

Table S1. Geographic distribution of *Fusarium* species obtained from durian die-back disease.

Province	Location of origin	Geographic Coordinates[1]			Isolate code	Group identification
		latitude	longitude	MAMSL[1]		
Chanthaburi	Makham district	12.63368	102.2421	35.8	**DCHA102***	**G15: *F. solani***
					DCHU104	G4: *F. solani*
					**DCHU107***	**G7: *F. solani***
					DCHU108	G1: *F. incarnatum*
					DCHU109	G7: *F. solani*
		12.63366	102.2421	36.6	DCHA202	G4: *F. solani*
					DCHA203	G3: *F. incarnatum*
					DCHA225	G4: *F. solani*
					DCHA226	G1: *F. incarnatum*
					DCHA406	G4: *F. solani*
		12.63318	102.2416	28.5	**DCHA408***	**G4: *F. solani***
					DCHA411	G4: *F. solani*
					**DCHA424***	**G17: *F. solani***
		12.63307	102.2419	33.3	DCHA505	G1: *F. incarnatum*
		12.63308	102.2424	43.7	DCHA604	G3: *F. incarnatum*
		12.63336	102.2425	34.9	DCHA701	G3: *F. incarnatum*
					DCHA702	G1: *F. incarnatum*
					DCHA703	G1: *F. incarnatum*
					**DCHA704***	**G1: *F. incarnatum***
	Khlung district	12.54329	102.2677	19.6	DCHA803	G3: *F. incarnatum*
					**DCHA805***	**G12: *F. mangiferae***
					DCHA806	G6: *F. incarnatum*
					2DCHA801	G10: *F. solani*
					2DCHA811	G12: *F. mangiferae*
					2DCHA817	G12: *F. mangiferae*

Province	Location of origin	Geographic Coordinates[1]			Isolate code	Group identification
		latitude	longitude	MAMSL[1]		
		12.54324	102.2678	19.7	DCHA902*	G8: *F. solani*
					**DCHA904***	**G16: *F. solani***
					DCHA905	G8: *F. solani*
		12.54319	102.2676	19.6	DCHA1002	G6: *F. incarnatum*
					DCHA1003	G10: *F. solani*
					DCHA1008	G1: *F. incarnatum*
					2DCHA1008	G2: *F. incarnatum*
					DCHU1010	G7: *F. solani*
		12.54326	102.2676	19.6	**DCHA1101***	**G11: *F. incarnatum***
					DCHA1103	G2: *F. incarnatum*
					DCHA1104	G1: *F. incarnatum*
		12.54313	102.2671	19.6	DCHA1204	G1: *F. incarnatum*
					DCHA1205	G1: *F. incarnatum*
					**DCHA1301***	**G2: *F. incarnatum***
					DCHA1302	G3: *F. incarnatum*
					DCHA1305	G3: *F. incarnatum*
					DCHA1403	G3: *F. incarnatum*
	Na Yai Am district	12.78327	101.8205	2.9	DCHA1504	G11: *F. incarnatum*
					DCHA1505	G2: *F. incarnatum*
		12.78322	101.8202	2.1	DCHA1606	G2: *F. incarnatum*
					**DCHA1607***	**G6: *F. incarnatum***
					DCHA1608	G2: *F. incarnatum*
		12.78342	101.8203	2.5	**DCHA1708***	**G10: *F. solani***
					DCHA1709	G2: *F. incarnatum*
					DCHA1713	G8: *F. solani*
		12.809	101.8364	11.5	**DCHA1801***	**G3: *F. incarnatum***
					DCHA1802	G1: *F. incarnatum*

Province	Location of origin	Geographic Coordinates[1]			Isolate code	Group identification
		latitude	longitude	MAMSL[1]		
		12.80881	101.8364	6.3	DCHA1901	G2: *F. incarnatum*
					DCHA1902	G2: *F. incarnatum*
		12.80875	101.8366	8.4	DCHA2002	G1: *F. incarnatum*
		12.80864	101.8368	6.4	DCHA2102	G1: *F. incarnatum*
					DCHA2103	G6: *F. incarnatum*
					DCHA2104	G2: *F. incarnatum*
					DCHA2105	G1: *F. incarnatum*
					DCHA2106	G2: *F. incarnatum*
Chumphon	Tha Sae district	10.77187	98.97505	99.4	DCHU206	G9: *F. solani*
		10.77259	98.97508	85.8	**DCHU303***	**G5: *F. solani***
		10.77289	98.97562	126.2	DCHU401	G1: *F. incarnatum*
					DCHU402	G5: *F. solani*
					**DCHU403***	**G13: *F. solani***
		10.77299	98.97555	100.1	DCHU504	G10: *F. solani*
		10.77995	98.97407	133.1	DCHU602	G3: *F. incarnatum*
					DCHU603	G1: *F. incarnatum*
					DCHU605	G2: *F. incarnatum*
					DCHU606	G1: *F. incarnatum*
	Pathio district	10.72001	99.26289	105.3	**DCHU801***	**G9: *F. solani***
	Phato district	9.881517	98.84368	64.6	DCHU904	G4: *F. solani*
		9.880598	98.84427	56.3	DCHU1201	G2: *F. incarnatum*
	Thung Tako district	10.06938	99.99303	80	DCHU1307	G1: *F. incarnatum*
					DCHU1308	G1: *F. incarnatum*
		10.06965	98.993	82.9	**DCHU1404***	**G14: *F. solani***
					DCHU1405	G1: *F. incarnatum*
					DCHU1407	G4: *F. solani*
		10.06829	98.992	81.3	DCHU1602	G3: *F. incarnatum*

Province	Location of origin	Geographic Coordinates[1]			Isolate code	Group identification
		latitude	longitude	MAMSL[1]		
	Mueang Chumphon district	10.51155	99.00991	41.3	DCHU1801	G9: *F. solani*
					DCHU1805	G14: *F. solani*
Trat	Khao Saming district	12.51849	102.4201	24.5	DTRA109	G3: *F. incarnatum*
					DTRA110	G3: *F. incarnatum*
		12.51841	102.4202	32	DTRA205	G2: *F. incarnatum*
					DTRA206	G4: *F. solani*
					DTRA209	G1: *F. incarnatum*

[1] Geographic location and elevation above mean sea level (AMSL) were determined using GPS status version 8.0.170.

[*] Representative isolate
