# Supplementary material for: Comprehensive Investigation of Die-Back Disease Caused by Fusarium in Durian
Source: Plants (Basel). 2023 Aug 24;12(17):3045. doi: 10.3390/plants12173045 (PMC10490359; doi:10.3390/plants12173045)
Supplement: Supplementary file 1 [file plants-12-03045-s001.zip › Table-S2.pdf]

Table S2. Analysis of distribution and relative abundance of *Fusarium* species.

**Location (province)**	**Number of *Fusarium* species (Isolate)**			**Total**
	F. incarnatum	***F. mangiferae***	***F. solani***	
Chanthaburi	39 (65.0%)	3 (5.0%)	18 (30.0%)	60 (100%)
Chumphon	10 (47.6%)	-	11 (52.4%)	21 (100%)
Trat	4 (80.0%)	-	1 (20.0%)	5 (100%)
Total	53 (61.63%)	3 (3.49%)	30 (34.88%	86 (100%)
